# Evaluation of the efficacy of the anti-ulcer oral mucosal protective agent RADoralex® in the prevention and treatment of radiation-induced oral mucosal reactions induced during treatment of nasopharyngeal carcinoma

**DOI:** 10.1080/15384047.2021.2013704

**Published:** 2022-01-06

**Authors:** Jun Yin, Jirui Xie, Jiawei Lin, Chengyin Weng, Shun Lu, Peng Xu, Shuo Zhang, Cheng Luo, Yecai Huang, Lu Li, Jinyi Lang, Mei Feng

**Affiliations:** a Department of Radiation Oncology, Sichuan Cancer Hospital & Institute, Sichuan Cancer Center, School of Medicine, University of Electronic Science and Technology of China, Chengdu, China; b Information Centre, Sichuan Cancer Hospital & Institute, Sichuan Cancer Center, School of Medicine, University of Electronic Science and Technology of China, Chengdu, China; c Department of Oncology, Guangzhou First People’s Hospital, Guangzhou, China; d Department of Radiation Oncology, The Third People’ S Hospital of Sichuan Province, Chengdu, China

**Keywords:** Antiulcer oral mucosal protectant, Radoralex®, nasopharyngeal carcinoma, radiation therapy, oral mucositis

## Abstract

The aim of this study was to evaluate the efficacy and safety of antiulcer oral mucosal protectant-RADoralex® in the prevention and treatment of radiation-induced oral mucosal reactions elicited during intensity-modulated radiation therapy (IMRT). for locally advanced nasopharyngeal carcinoma (NPC). A total of 90 patients with locally advanced NPC who developed post-treatment grade 1 oral mucositis were selected for this study. They were randomly assigned to the experimental group (n = 44) treated by mouth rinsing with the RADoralex® during radiochemotherapy and the control group (n = 43) treated by mouth rinsing with sodium bicarbonate solution, and the patients’ oral mucosal conditions, quality of life, weight change and oral pain levels were analyzed. The incidence of Common Terminology Criteria for Adverse Events (CTCAE) v4.0 grade 2 and grade 3 oral mucositis were significantly lower in the experimental group than in the control group. Compared to the control group, the time to progression, and the time from the end of treatment to oral mucosa healing in the experimental group was significantly shorter. The experimental group lost 8.66 ± 3.543% of their body weight during treatment period, while the control group lost 12.53 ± 4.284% (*p* < .001). From the beginning the 3^rd^ week of treatment to the 2^nd^ week after the end of treatment, the Oral Mucositis Assessment Scale (OMAS) scores were lower in the experimental group than in the control group (*p* < .05). RADoralex® significantly reduced the incidence and severity of oral mucositis in patients with locally advanced NPC during radiochemotherapy, delayed the progression of oral mucositis.

## 1. Introduction

Nasopharyngeal carcinoma (NPC) is a major type of malignant otorhinolaryngologic neoplasm in China.^1^ The incidence rate of NPC decreases progressively from south to north in China. NPC is particularly prevalent in the southern part of China and in the Guangxi and Guangdong provinces.^2^ At present, radiation therapy is the preferred treatment for NPC.^3^ With the continuous updating of radiotherapy equipment and treatment technology, intensity-modulated radiation therapy (IMRT) has now become the main treatment for NPC.^4^ Compared with conventional radiotherapy, IMPT offers better protection of normal tissues and significantly reduces the acute phase response and late phase reactions.^5^ However, the nasopharynx is anatomically located in close proximity to the oropharynx and oral cavity. Moreover, most patients already suffer locally advanced NPC at the time they seek treatment and thus need to undergo concurrent chemotherapy. As the result, oral mucositis is elicited or aggravated.^6^ According to previous reports, the incidence of oral mucositis in patients with NPC treated with radiochemotherapy is 85–100%, while the incidence of grade 3 or higher oral mucositis is 28–35%.^7,8^

The main manifestations of oral mucositis are discomfort or pain in the oral cavity or throat, xerostomia or parageusia. In severe cases, the inability to eat and even the necessity to withdraw from treatment. To date, various interventions have been evaluated in clinical practice to treat and alleviate radiation-induced oral mucosal reactions. However, no one measure has been found to be significantly superior in the treatment or alleviation of treatment-related oral mucositis.^7^

Common measures used in our hospital to prevent and treat oral mucositis caused by radiotherapy include mouth rinsing with sodium bicarbonate solution and Kangfuxin solution, inhalation of nebulized solution (such as a mixture of lidocaine, dexamethasone and chymotrypsin), anti-infective therapy, analgesic therapy, nutritional support treatment and cytoprotection. Such measures ensure that patients with NPC will receive appropriate symptomatic treatments after the occurrence of treatment-related oral mucosal reactions, and thus successfully complete their treatments. Nevertheless, most patients with NPC will still experience radiation-induced oral mucosal reactions to varying degrees during radiotherapy.

This study was intended to evaluate the efficacy and safety of antiulcer oral mucosal protectant-RADoralex® in the prevention and treatment of radiation-induced oral mucosal reactions induced during treatment of nasopharyngeal carcinoma due to IMRT.

## 2. Materials and methods

### 2.1 Experimental design and patient enrollment

This study was designed to determine superior efficacy of RADoralex. 90 patients with locally advanced NPC who received radiochemotherapy and developed post-treatment grade 1 oral mucositis between December 2015 and November 2016 at Guangzhou First Municipal People’s Hospital, China, and our hospital were selected for this study. This study was conducted according to the guidelines set forth in the Declaration of Helsinki and was approved by the Ethics Committee of Sichuan Cancer Hospital. All 90 patients signed an informed consent document.

### 2.2 Inclusion and exclusion criteria

Patients were eligible for inclusion in this study if they had the 1) definitive diagnosis of NPC by histopathologic examination; 2) *an* age ≥18 years and ≤80 years; 3) Karnofsky Performance Status (KPS score) equal to or greater than 70 points; 4) concurrent radiochemotherapy (using IMRT); 5) treatment-related grade 1 oral mucosal reaction during radiochemotherapy; 6) had the following routine blood test results: hemoglobin level ≥100 g/L, platelet count ≥75 × 10^9^/L, white blood cell count ≥3.0 × 10^9^/L, and absolute neutrophil count ≥1.5 × 10^9^/L; and 7). The patient exclusion criteria were as follows: hypersensitivity to any of the ingredients in the RADoralex® or have an obvious allergic constitution. Subjects were randomized using a computer-generated random number codes. Subjects were stratified according to the treatment center and then randomly assigned in a 1:1 ratio to receive either RADoralex® treatment (experimental group, n = 44, 1 case withdrawn) or sodium bicarbonate treatment (control group, n = 43, 2 cases withdrawn). The investigation lasted 14 weeks to improve oral mucositis in patients with locally advanced NPC during radiochemotherapy.

### 2.3 Intervention

Both groups were routinely treated according to the following principles. 1) Both groups received conventional health education, routine care and relevant treatments: mouth rinsing with clean water, Kangfuxin solution, nebulized inhalation (a mixture of lidocaine, dexamethasone, and chymotrypsin), anti-infective therapy, analgesic therapy, nutritional support therapy, and cytoprotective agents. 2) Obtain pharyngeal swabs as soon as signs of infection appear and then promptly administer empirical anti-infective therapy. The groups were then given targeted anti-infective treatments based on the results of the throat swab culture and drug susceptibility tests. 3) To treat oral and pharyngeal pain, the groups received analgesics according to the World Health Organization (WHO) three-step analgesic ladder for cancer pain management. 4) Radiotherapy was temporarily discontinued if patients developed severe radiation-induced oral mucosal reactions (grade 3 or higher). Moreover, intravenous anti-infective treatment was fortified.

The experimental group received RADoralex® in addition to the conventional treatment described above. The treatment procedure was as follows. 1) For patients with early mild oral mucosal reactions (grade 1), patients rinsed their mouths with 5–8 mL of the protectant 3 times/day for 1–2 min. Spit out the liquid after each rinse. 2) In patients with moderate to severe oral mucosal reactions (≥grade 2), the patients rinsed their mouths with 15–20 mL of the protectant 4–5 times/day for 1–2 min. The liquid was spit out after each rinse. 3) If the patient suffered concurrent ulcers in deep nasopharyngeal regions such as the posterior pharyngeal wall, he/she was instructed to swallow an appropriate amount of protectant until the radiotherapy was completed and the radiation-induced oral mucosal injuries have healed.

The control group received sodium bicarbonate solution in addition to the conventional treatments described above. The treatment procedure was as follows. 1) For patients with early mild oral mucosal reactions (grade 1), patients rinsed their mouths with sodium bicarbonate solution (1–2 min each time) 3 times/day. The liquid was spit out after each rinse. 2) In patients with moderate to severe oral mucosal reactions (≥grade 2), the patients rinsed their mouths with sodium bicarbonate solution (1–2 min each time) 4–5 times/day spit out the liquid after each rinse. 3) If a patient suffered concurrent ulcers in deep nasopharyngeal regions such as the posterior pharyngeal wall, he/she was instructed to swallow an appropriate amount of sodium bicarbonate solution until the radiotherapy was completed and the radiation-induced oral mucosal injuries were healed.

Compliance was defined as completing 100% of the treatment. Patients who did not meet the requirements were excluded.

### 2.4 The primary endpoint

The primary endpoint was the incidence of oral mucosal reactions of the 3rd degree or higher degree oral mucosal reactions. According to previous reports and our clinical experience, the incidence of 3rd degree or higher oral mucosal reactions in the control group (sodium bicarbonate solution group) and the experimental group (RADoralex® group) was estimated to be 40% and 10%, respectively.

### 2.5 Radiochemotherapy

All patients underwent radical radiationtherapy during which IMRT technique was employed. The radiation doses were delivered as follows: the gross tumor volume received a total dose of 66–74 Gy (fractional dose: 2.1–2.3 Gy), the positive lymph nodes received a total dose of 60–70 Gy (fractional dose: 2.0–2.2 Gy), the high-risk clinical target volume received a total dose of 60–66 Gy (fractional dose: 1.8–2.0 Gy), the low-risk clinical target volume received a total dose of 54–60 Gy (fractional dose: 1.8–2.0 Gy), and total dose received in the cervical lymphatic drainage area was 50 Gy (fractional dose: 1.8–2.0 Gy). The patients received IMRT 5 days per week with 2 days off. Chemotherapy comprised cisplatin (75 mg/m^2^) alone, and one cycle of cisplatin-based chemotherapy lasted 21 days.

### 2.6 Evaluation criteria

After the start of radiotherapy, the patient was examined daily by the physician. The patient’s oral mucosal condition and the occurrence of adverse events were recorded daily. In addition, the patients’ quality of life scores and body weights were recorded weekly.

The main evaluation indices were as follows: 1) Common Terminology Criteria for Adverse Events (CTCAE) v4.0, used to objectively classify the radiation-induced oral mucosal reactions; 2) Oral Mucositis Assessment Scale (OMAS), used to grade the severity of ulcers; the following were the OMAS ulceration score criteria: Grade 0 indicates that there is no lesion; Grade 1 indicates that the lesion is < 1 cm^2^; Grade 2 indicates that the lesion is between 1 and 3 cm^2^; and Grade 3 indicates that the lesion is > 3 cm^2^; and 3) European Organization for Research and Treatment of Cancer (EORTC) Quality of Life Questionnaire-C30 (QLQ-C30) version 3.0.

The secondary evaluation indices included 1) oral and pharyngeal pain scores, 2) weight change before and after radiotherapy, and 3) time from the end of radiotherapy to the healing of oral mucosal injuries. If a patient developed intolerable toxicity, no longer agreed to continue to participate in the clinical study, suffered disease progression, died, or had to terminate treatment due to some other reasons, he/she was withdrawn from the study.

### 2.7 Follow-up

The follow-up period begins after the patient is discharged from the hospital at the end of radiotherapy. The time elapsed from the end of radiotherapy to oral mucosal injury/healing (or patient death) was recorded by weekly hospital visits or telephone follow-ups.

### 2.8 Statistical analysis

In determining the required sample size to achieve 1-β = 0.80 and α = 0.05, the sample size was first calculated using Power and Sample Size (PASS) V3.0 software. It was estimated that the experimental and the control groups should each include at least 38 subjects. Subsequently, the sample size was adjusted to 43 subjects per group based on an anticipated dropout rate of 10%. Considering the general requirements for randomization, we made further adjustment to include 45 subjects per group (90 subjects in total for both groups). All statistical analyses were performed using SPSS 24.0 statistical software. The following statistical methods were employed: if the measurement data conformed to a normal distribution and showed homogeneity of variance, a t test was performed, and the mean, standard deviation, and 95% confidence interval (CI) were presented. Otherwise, the rank sum test was employed. Count data were analyzed using the chi-square test, and the frequency and percentage were calculated. The significance level of the two-tailed test was set at 0.05 with a 95% CI.

## 3. Results

### 3.1 General information

**Figure 1.**
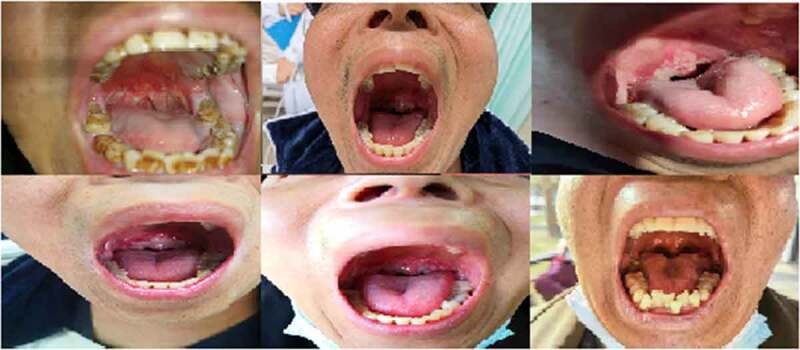
Mucositis sample from lingual margin, soft palate, posterior molar area and pharyngeal wall.

A total of 90 patients were included in this study, 45 of whom were included in the experimental group and 45 in the control group. Three patients withdrew from the study (2 patients discontinued the treatment due to intolerance and 1 patient refused to continue the investigational drug during the treatment period). Therefore, a total of 87 patients were subjected to the final statistical analysis. There was no heterogeneity between the experimental group and the control group in terms of gender, age, stage, and KPS score (*p* > .05, Table 1). There were no cases of gastric tube placement in either group. Figure 1 was a representative photograph of mucositis observed according to CTCAE 4.0 and OMAS.

**Table 1.** Demographic and baseline characteristics of valid cases

Baseline characteristics	Experimental group	Control group	*t*/*x^2^* -value	*P*-value
Gender			0.630	0.427
Male	34 (77.3)	30 (69.8)		
Female	10 (22.7)	13 (30.2)		
Age (years) mean ± SD	47.55 ± 10.817	46.42 ± 10.128	0.501	0.617
KPS score			0.665	0.717
80 points	8 (18.2)	6 (20.9)		
90 points	34 (77.3)	36 (74.4)		
100 points	2 (4.5)	1 (4.7)		
T stage			2.642	0.450
1	3 (6.8)	6 (14.0)		
2	11 (25.0)	6 (14.0)		
3	12 (27.3)	14 (32.6)		
4	18 (40.9)	17 (39.5)		
N stage			1.746	0.418
1	7 (15.9)	5 (11.6)		
2	27 (61.4)	32 (74.4)		
3	10 (22.7)	6 (14.0)		
Clinical stage			1.381	0.240
3	18 (40.9)	23 (53.5)		
4	26 (59.1)	20 (46.5)		
Neoadjuvant chemotherapy			2.529	0.112
yes	28(63.6)	34(70.1)		
no	16(36.4)	9(20.9)		

All data are presented as n (%) unless otherwise stated.

### 3.2 Therapeutic efficacy

#### 3.2.1 Incidence rates of oral mucositis and the time to progression

**Figure 2.**
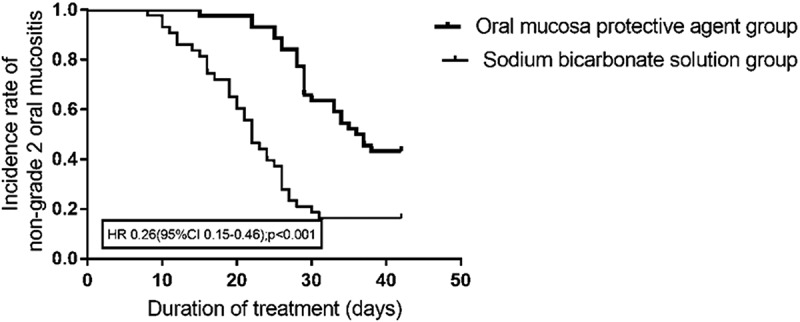
The incidence rates of grade 2 oral mucositis.

**Figure 3.**
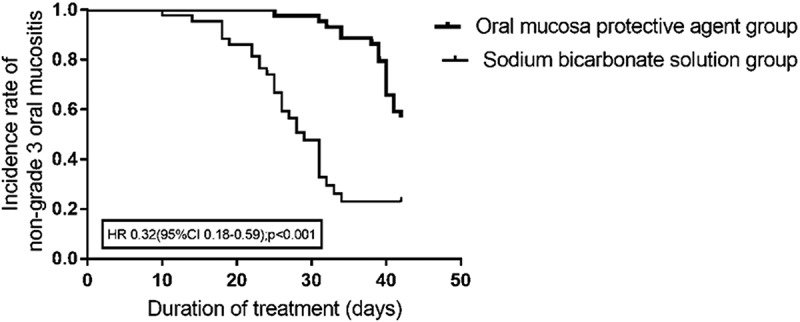
The incidence rates of grade 3 oral mucositis.

The incidence rates of grade 2 and grade 3 oral mucositis were significantly lower in the experimental group than in the control group (56.8% vs 83.7%, *p* = .006; 43.2% vs 67.4%, *p* = .023; Table 2, Figure 2, Figure 3). The time to progression (length of time for oral mucositis to progress from grade 1 to grade 2, from grade 2 to grade 3 and from grade 1 to grade 3, length of time from low to high grade dates for oral mucositis) was increased in the experimental group compared to the control group (*p* < .001, *p* = .016, *p* < .001) (Table 3). In addition, the time from the end of treatment to the healing of the oral mucosa was reduced in the experimental group (*p* = .026) (Table 3).

**Table 2.** The incidence rates of oral mucositis

Grade (CTCAE4.0),n (%)	Experimental Group	Control group	*x^2^*- value	*P*-value
2	25 (56.8)	36 (83.7)	7.512	0.006
3	19 (43.2)	29 (67.4)	5.175	0.023
4	1 (2.3)	1 (2.3)	0.000	0.987
≥3	20 (45.5)	30 (69.8)	5.259	0.022

**Table 3.** Time to progression and healing time (days) (CTCAE4.0)

	Experimental group	Control group	*t* value	P-value
Grade 1 to grade 2	19.44 ± 5.895	11.36 ± 5.953	5.233	<0.001
Grade 2 to grade 3	7.95 ± 3.979	5.38 ± 3.144	2.490	0.016
Grade 1 to grade 3	27.26 ± 6.063	16.66 ± 6.195	5.850	<0.001
Healing time (days)	26.83 ± 17.206	34.67 ± 15.022	−2.267	0.026

From the 3^rd^ week of treatment to the 6^th^ week after the end of treatment, the OMAS scores of the experimental group were significantly lower than those of the control group (*p* < .05, Table 4).

**Table 4.** Comparison of OMAS scores between the two groups

	Experimental group	Control group	*t-* value	*P*-value
1^st^ week of treatment	0.009 ± 0.4210	0.014 ± 0.5160	−0.482	0.631
2^nd^ week of treatment	0.291 ± 0.4102	0.344 ± 0.3065	−0.685	0.495
3^rd^ week of treatment	0.350 ± 0.4526	0.930 ± 0.4974	−5.693	<0.001
4^th^ week of treatment	0.423 ± 0.4650	1.237 ± 0.5827	−7.215	<0.001
5^th^ week of treatment	0.568 ± 0.4564	1.447 ± 0.6092	−7.623	<0.001
At the end of treatment	0.641 ± 0.6318	1.674 ± 0.5888	−7.889	<0.001
1 week after the end of radiotherapy	0.627 ± 0.6507	1.326 ± 0.5790	−5.284	<0.001
2 weeks after the end of treatment	0.395 ± 0.4430	0.856 ± 0.4436	−4.843	<0.001
3 weeks after the end of treatment	0.232 ± 0.3422	0.577 ± 0.3709	−4.510	<0.001
4 weeks after the end of treatment	0.123 ± 0.2836	0.293 ± 0.2482	−2.978	0.004
5 weeks after the end of treatment	0.064 ± 0.1869	0.219 ± 0.2383	−3.379	0.001
6 weeks after the end of treatment	0.023 ± 0.8860	0.107 ± 0.2558	−2.062	0.042
7 weeks after the end of treatment	0.027 ± 0.1107	0.070 ± 0.1684	−1.394	0.167
8 weeks after the end of treatment	0.018 ± 0.7240	0.033 ± 0.8650	−0.841	0.403

#### 3.2.2 Oral pain, body weight, and quality of life

Compared to the weight before treatment, the experimental group had lost 8.66 ± 3.543% during the treatment period, while the control group lost 12.53 ± 4.284% (*t* = −4.603, *p* < .001). The quality of life scores (QLQ-C30) was lower in the experimental group than in the control group from the beginning of the 2nd week of treatment to the 3rd week after the end of treatment ((*p* < .05, Table 5). From week 3 of treatment until week 2 after the end of treatment, the experimental group had lower oral and throat pain scores than the control group(*p* < .05, Table 6).

**Table 5.** Comparison of QLQ-C30 (V3.0) scores between the two groups

	Experimental group	Control group	*t-*value	*P*-value
1^st^ week of treatment	48.77 ± 6.664	49.88 ± 8.592	−0.675	0.502
2^nd^ week of treatment	51.84 ± 9.487	56.37 ± 8.486	−2.346	0.021
3^rd^ week of treatment	54.57 ± 11.403	70.58 ± 14.144	−5.820	<0.001
4^th^ week of treatment	60.09 ± 12.201	79.23 ± 14.244	−6.737	<0.001
5^th^ week of treatment	59.89 ± 13.833	88.65 ± 13.406	−9.846	<0.001
At the end of treatment	61.80 ± 15.210	96.00 ± 10.623	−12.135	<0.001
1 week after the end of treatment	57.14 ± 13.438	84.79 ± 10.580	−10.649	<0.001
2 weeks after the end of treatment	54.43 ± 12.433	72.95 ± 9.504	−7.793	<0.001
3 weeks after the end of treatment	52.57 ± 10.256	58.67 ± 9.670	−2.856	0.005
4 weeks after the end of treatment	50.86 ± 8.399	53.70 ± 8.967	−1.522	0.132
5 weeks after the end of treatment	49.84 ± 8.710	49.88 ± 8.669	−0.023	0.982
6 weeks after the end of treatment	46.52 ± 8.001	46.79 ± 8.171	−0.155	0.878
7 weeks after the end of treatment	45.36 ± 7.607	45.49 ± 7.116	−0.079	0.937
8 weeks after the end of treatment	45.09 ± 7.511	44.63 ± 6.291	0.311	0.756

**Table 6.** Comparison of oral pain scores between the two groups

	Experimental group	Control group	*t-*value	*P*-value
1^st^ week of treatment	0.30 ± 0.462	0.30 ± 0.638	−0.058	0.954
2^nd^ week of treatment	1.45 ± 0.926	1.81 ± 1.075	−1.672	0.098
3^rd^ week of treatment	1.80 ± 0.930	2.49 ± 0.935	−3.465	0.001
4^th^ week of treatment	2.05 ± 1.077	3.44 ± 0.934	−6.455	<0.001
5^th^ week of treatment	2.09 ± 0.858	4.30 ± 0.773	−12.626	<0.001
At the end of treatment	2.23 ± 0.937	4.51 ± 0.736	−12.630	<0.001
1 week after the end of treatment	1.80 ± 1.002	3.09 ± 0.684	−7.041	<0.001
2 weeks after the end of treatment	1.39 ± 0.970	1.91 ± 0.996	−2.471	0.015
3 weeks after the end of treatment	0.93 ± 0.818	1.07 ± 0.768	−0.810	0.420
4 weeks after the end of treatment	0.66 ± 0.745	0.67 ± 0.680	−0.100	0.920
5 weeks after the end of treatment	0.52 ± 0.698	0.53 ± 0.702	−0.081	0.936
6 weeks after the end of treatment	0.34 ± 0.568	0.33 ± 0.522	0.131	0.896
7 weeks after the end of treatment	0.27 ± 0.499	0.28 ± 0.591	0.657	0.957
8 weeks after the end of treatment	0.27 ± 0.451	0.21 ± 0.514	0.612	0.542

#### 3.2.3 Safety

No serious adverse events, such as allergies, anaphylactic shock were observed in either group. Adverse events were observed in 42 of 44 patients (95.5%) in the experimental group as well as 41 of the 43 patients (95.3%) in the control group (*p* = .981). The most common adverse events (>50%) were decreased white blood cell count (86.4% vs 90.7%, *p* = .526), hemoglobin level (59.10% vs 55.89%, *p* = .757), and platelet count (22.70% vs 27.99%, *p* = .578). The above adverse events were mainly caused by radiochemotherapy. There was no statistically significant difference in the incidence of adverse events between the two groups.

## 4. Discussion

Oral mucositis occurs in virtually 100% of the patients with head and neck cancers who have received concurrent radiotherapy and chemotherapy.^9^ Oral mucositis may reduce the tolerance of patients to radiotherapy, thereby decreasing the total dose of radiotherapy.^4^ Radiotherapy is the main treatment for NPC. Patients with locally advanced NPC undergo concurrent chemotherapy, which further increases the incidence of oral mucositis. Moreover, oral mucositis may interrupt treatment or even cause patients to abandon treatment, which accelerates the proliferation of residual tumor cells, results in tumor recurrence or metastasis, and reduces the long-term survival rate of patients.^10^ Therefore, the prevention and treatment of oral mucositis are of great clinical significance.

A number of previous studies have explored the pathogenic mechanism, preventive measures, and treatments for radiation-induced oral mucositis. Reactive oxygen species (ROS) produced during radiotherapy activate nuclear transcription factor NF-κB (nuclear factor kappa B), proinflammatory cytokines (such as tumor necrosis factor-α and interleukin-6) and metabolic byproducts that promote apoptosis, aggravate tissue damage, cause bacterial, fungal, and viral infections, and aggravate radiochemotherapy-induced oral mucositis in patients with NPC.^11,12^

Alpha-lipoic acid is a strong antioxidant. It increases the level of glutathione in normal tissues by reacting with free radicals,^13^ thereby preventing radiotherapy-induced normal tissue damage in patients with head and neck tumors.^14^ Radiotherapy induces the necrosis or apoptosis of epithelial cells and abolishes the compensatory effect caused by cell proliferation, resulting in epithelial destruction.^15^ Basic fibroblast growth factor contributes to the proliferation of various cell types (epithelial cells, fibroblasts, vascular smooth muscle cells, and keratinocytes) and the formation of granulation tissue,^16^ thus protecting the mucosa from radiotherapy-induced damage.^17^ In addition, randomized clinical studies have demonstrated that honey,^18^ black mulberry,^19^ certain botanical ingredients, and proprietary Chinese medicine preparations (such as Zataria extract,^20^ Shuanghua Baihe tablets,^21^ and Kangfuxin Solution^22^) prevent radiochemotherapy-induced oral mucositis in patients with head and neck tumors. However, most of the above-mentioned studies employed placebo or normal saline as controls or blank controls, and thus the efficacy of the investigational drugs could not be accurately assessed. In addition, all of the above studies employed drug-based treatments, which not only had slow-acting effects but also caused certain side effects. After radiotherapy, the oral mucosa becomes ulcerated and susceptible to infection by bacteria, fungi, and other microorganisms. Physically blocking the invasion of pathogens on the oral mucosa surface is expected to prevent the occurrence and progression of radiotherapy- associated oral mucositis. However, to date, no such studies have been reported.

In this study, patients in the control group rinsed their mouths with sodium bicarbonate solution. Moreover, the patients enrolled in this study all suffered locally advanced NPC and showed little heterogeneity in the radiotherapy target range, radiotherapy dose, and concurrent chemotherapy. Therefore, the results of this study more closely reflected the true efficacy of the drug. The RADoralex® is a pseudoplastic fluid when diluted with pure water. It covers the mucosa and forms a thin sticky coating that acts as a physical barrier, blocking the invasion of pathogenic bacteria.

In addition, the RADoralex® creates a microenvironment that promotes self-healing of damaged mucosal epithelial cells and can prevent and treat oral mucositis and mucosal ulcers. Compared to other oral medications, the RADoralex® has fewer side effects and exerts a faster effect. This study used multiple scales to evaluate patients’ oral mucositis, oral pain levels, and quality of life. The results demonstrated that the RADoralex® significantly reduced the incidence and severity of radiochemotherapy-induced oral mucositis in patients with locally advanced NPC, delayed the progression of oral mucositis, promoted the healing of the oral mucosa, and relieved oral and throat pain. In addition, the RADoralex® reduced weight loss during treatment and improved the quality of life of patients.

No serious adverse events were observed in this study. There were no statistically significant differences in the incidence and severity of adverse events between the two groups. Therefore, RADoralex® can be used clinically to reduce and delay the occurrence of oral mucositis in patients with locally advanced NPC during radiochemotherapy and enhance patient tolerance to treatments. However, patients in the control group with N2 stage were higher than those in the trial group, and the increase in the extent of cervical lymph node radiation therapy would result in an increase in the dose and volume of irradiated oral cavity and larynx, which would lead to the development of oropharyngeal mucositis and may affect the findings of this study.

Limitations of this study included the small sample size and short follow-up period. The long-term toxicity of the drug and the long-term prognosis of the patients were not examined. Furthermore, there was little data collection on the dosage of analgesics and other adjuvant medications. We also did not have data on the latency of mucositis or the duration of mucositis after the radiotherapy. In addition, we did not distinguish the between oral mucositis caused by radiotherapy and mucositis caused by chemotherapeutic agents. In the future, large randomized controlled studies with stratified analysis based on T and N staging should be considered, and the induction chemotherapy as an independent factor in the outcome analysis and longer follow-up periods are needed to further understand the toxic response and efficacy of RADoralex® at different stages. Additionally, we only observed the recent efficacy of RADoralex® in protecting the oral mucosa and reducing the mucosal reaction of patients, and its effect on local control (LC), locoregional control (LRC) and overall survival (OS) should be further analyzed in the long-term follow-up of both groups.

## 5. Conclusion

RADoralex® reduces the incidence and severity of oral mucositis in patients with locally advanced NPC during radiochemotherapy, delays the progression of oral mucositis, promotes healing of the oral mucosa, and improves the quality of life of patients.

## Data Availability

The datasets used or analyzed during the current study are available from the corresponding author on reasonable request.
